# MicroRNA-125b-5p Correlates With Prognosis and Lung Adenocarcinoma Progression

**DOI:** 10.3389/fmolb.2021.788690

**Published:** 2022-02-03

**Authors:** Lin Tang, Yixiao Yuan, Haoqing Zhai, Juan Wang, Dahang Zhang, Huasu Liang, Yulin Shi, Lincan Duan, Xiulin Jiang

**Affiliations:** ^1^ The Department of Thoracic Surgery, The Third Affiliated Hospital of Kunming Medical University, Kunming, China; ^2^ Department of Graduate School of Kunming Medical University, Kunming, China; ^3^ Key Laboratory of Animal Models and Human Disease Mechanisms of Chinese Academy of Sciences and Yunnan Province, Kunming Institute of Zoology, Kunming, China; ^4^ Kunming College of Life Science, University of Chinese Academy of Sciences, Beijing, China

**Keywords:** miRNA-125b-5p, non-small cell lung cancer, diagnostics, biomarkers, cell proliferation, cell migration, cell apoptosis

## Abstract

A growing number of studies have focused on investigating microRNAs as crucial regulators in the progression of multiple cancer types. Nevertheless, the biological effects and immunological role of miR-125b-5p in non-small cell lung cancer (lung adenocarcinoma, LUAD) have not been determined. The present study aimed to examine the function of miR-125b-5p on cell proliferation and the outcomes of LUAD patients. We utilized diverse public databases in the analysis of the expression, prognosis, diagnostic value, and immune role of miR-125b-5p in non-small cell lung cancer. The growth curve, colony formation, flow cytometry, and Transwell and invasion assays were utilized to determine the function of miR-125b-5p in LUAD progression. In this study, we found that miR-125b-5p was decreased in LUAD and correlated with poor prognosis. Pathway analyses revealed that miR-125b-5p was mainly involved in cell proliferation and immune regulation. Moreover, *in vitro* experiments indicated that the overexpression of miR-125b-5p significantly inhibited cell proliferation, migration, and invasion and induced cell apoptosis of LUAD. Finally, we discovered that miR-125b-5p correlated with immune cell infiltration. In summary, these results demonstrated that miR-125b-5p serves as a prognostic marker and a therapeutic target for LUAD.

## Introduction

Lung cancer remains the most common deadly disease, with an estimated 2.09 million new cases and 1.76 million deaths each year (Rivera and Wakelee, 2016). Non-small cell lung cancers (NSCLCs), the most common lung cancers, are known to have diverse pathological features. As the most common histologic subtype of lung cancer, lung adenocarcinoma (LUAD) usually results in a lower 5-year survival rate in lung cancer (Schwartz and Cote, 2016; Liang et al., 2021). Although there are many diverse treatments, including chemoradiotherapy, targeted therapy, and immunotherapy, the prognosis of LUAD remains poor because it lacks effective diagnostic markers. Therefore, it is urgent to further uncover specific prognostic prediction methods for the diagnosis and treatment of lung cancer.

MicroRNAs (miRNAs) are non-coding RNAs that often inhibit gene expression at the posttranscriptional level. Mounting evidence has demonstrated that miRNAs play an important role in modulating lung cancer cell proliferation, invasion, migration, and apoptosis (Iqbal et al., 2019). For instance, Qu et al. found that miR-133b was downregulated in renal cell carcinoma cell lines. Overexpression of miR-133b significantly inhibits cell proliferation, migration, and invasion by targeting MMP9 in renal cell carcinoma (Wu et al., 2014). Furthermore, Wu et al. showed that miR-125b was decreased in bladder cancer specimens. An elevated level of miR-125b inhibits cell growth and migration and induces cell cycle arrest in the G1 phase, with a further study showing miR-125b targeting SphK1 and inhibiting bladder cancer progression (Zhao et al., 2015). Previous studies have shown that miR-125b plays a tumor suppressor role in breast cancer (Wang et al., 2020), cervical cancer (Sun et al., 2019), colorectal cancer (Yagi et al., 2019), and prostate cancer (Zedan et al., 2019). However, the underlying mechanisms of how miR-125b-5p regulates LUAD progression and metastasis remain elusive, which enhances the significance of the findings of the present study.

In this study, we found that miR-125b-5p was notably downregulated in LUAD tissues and cells and inhibited the proliferative and metastatic phenotypes in LUAD cell lines. Significantly, we demonstrated that miR-125b-5p is correlated with immune cell infiltration. In summary, these results showed that miR-125b-5p is a potential molecular marker for poor prognosis in LUAD and provides potential diagnostic and immunotherapeutic biomarkers for LUAD in the future.

## Methods

### Expression, Prognosis, and Clinical Information Analysis

We mainly used The Cancer Genome Atlas (TCGA) and the Kaplan–Meier plotter (http://kmplot.com/analysis/) (Nagy et al., 2021) to analyze the expression, prognosis, and clinical information of miR-125b-5p in cancers.

### Gene Set Enrichment and Immune Infiltration Analysis

We utilized the clusterProfiler package for the analysis of the potential signaling pathway and molecular function of the miR-125b-5p target gene in LUAD (Subramanian et al., 2005; Yu et al., 2012). We used the GSVA package in R to examine the LUAD immune infiltration of 24 tumor-infiltrating immune cells in tumor samples through single-sample gene set enrichment analysis (ssGSEA) (Bindea et al., 2013; Hänzelmann et al., 2013). The correlation between miR-125b-5p and the infiltration levels of immune cells was analyzed with Spearman’s correlation, and the immune cells with different expression groups of miR-125b-5p were analyzed using the Wilcoxon rank-sum test.

### Cell Culture, Constructs, and Transfection

The BEAS-2B cell line was purchased from the Cell Bank of Kunming Institute of Zoology and cultured in BEGM media (CC-3170; Lonza, Basel, Switzerland). Lung cancer cell lines, including A549, H1299, H358, HCC827, SPC-A1, H1650, and H1975, were purchased from Cobioer (Nanjing, China), with STR document, and were cultured in RPMI-1640 medium (Corning, Corning, NY, United States) supplemented with 10% fetal bovine serum (FBS) and 1% penicillin/streptomycin. The normal control (NC), miR-125b-5p mimics, and miR-125b-5p inhibitors were purchased from RiboBio (Guangzhou, China). Cells were transfected with the indicated miRNA mimics or NC using Lipofectamine 2000 (Invitrogen, Carlsbad, CA, United States) and then collected for various experiments.

### Cell Proliferation and Colony Formation Assays

Cell proliferation and colony formation assays were performed as previously documented (Xiong et al., 2021). Briefly, for the cell proliferation assay, the indicated cells were plated into 12-well plates at a density of 1.5 × 10^4^, and the cell numbers were subsequently counted each day using the automatic cell analyzer Countstar (IC 1000; Shanghai Ruiyu Biotech Co., Shanghai, China). For the colony formation assay, the indicated cells were seeded in a 6-well plate (cat. 703001; NEST, Jiangsu, China) with 600 cells per well supplemented with 2 ml cell culture medium. The cell culture medium was changed every 3 days for 2–3 weeks, and then the indicated cells were fixed with 4% paraformaldehyde (PFA) and stained with 0.5% crystal violet. For the tumor sphere formation assay, the indicated cells were plated in an ultralow-attachment 6-well plate (cat. 3471; Corning), cultured in serum-free DMEM/F12 supplemented with B27 (cat. 2309544; Gibco, Waltham, MA, USA), 20 ng/ml epidermal growth factor (EGF) and 20 ng/ml basic fibroblast growth factor (bFGF), and 4 μg/ml heparin. From 10 to 14 days after culture, the spheres were photographed and counted using a Nikon inverted microscope (Ti-S).

### Cell Migration Assay

Cell migration assays were performed as previously documented (Xiong et al., 2021). Briefly, to produce a wound, the monolayer cells on the 6-well plate were scraped in a straight line with pipette tips. The plate was then washed with PBS to remove detached cells. Photographs of the scratch were taken at indicated time points using the Nikon inverted microscope (Ti-S). Gap width was calculated using GraphPad Prism software. For the Transwell assay, 2.5 × 10^4^ cells in 100 μl serum-free medium were plated in a 24-well plate chamber insert (cat. 3422; Corning Life Sciences) with a medium containing 10% FBS at the bottom of the insert. The cells were incubated for 24 h and then fixed with 4% PFA for 20 min. After washing, the cells were stained with 0.5% crystal violet.

### Flow Cytometry Assay

The Annexin V FITC Apoptosis Detection Kit I (556547; BD, Shanghai, China) was used to evaluate cellular apoptosis according to the manufacturer’s instructions. For cell flow cytometry experiments, the indicated cells were digested and washed with PBS twice and then fixed in 75% alcohol overnight at −20°C. The fixed cells were washed three times and then stained with propidium iodide (PI) buffer at room temperature for 30 min in the dark. The cell cycle was then analyzed using the FACSAria SORP machine (BD, San Diego, CA, United States).

### Real-Time RT-PCR Assay

Quantitative real-time PCR (qRT-PCR) assay was performed as documented (Jiang et al., 2018). The primer sequences were as follows: miR-125b-5p primer: TCC​CTG​AGA​CCC​TAA​CTT​GTG​A; U6-F: GGT​CGG​GCA​GGA​AAG​AGG​GC; and U6-R: GCT​AAT​CTT​CTC​TGT​ATC​GTT​CC. The expression quantification was obtained with the 2^−ΔΔCt^ method.

### Western Blot Assay

The Western blot and immunohistochemistry staining assay was performed as documented. Briefly, cell lysates were collected, Western blot was performed, and the primary antibody was incubated overnight, followed by secondary antibody incubation. Finally, develop using instruments. Detailed information of the antibodies utilized in the study is as follows:

**Table:** 

Antibody name	Catalog no.	Dilution	Supplier	Species
β-actin	60008-1-1g	1:5,000	Proteintech	Mouse
PARP	9542S	1:1,000	CST	Rabbit
Bcl-2	15071S	1:500	CST	Mouse
Bax	ab77566	1:1,000	Abcam	Mouse

### Statistical Analysis

Significance of the data between two experimental groups was determined by Student’s t-test, and multiple group comparisons were analyzed using one-way ANOVA. Values of *p < 0.05, **p < 0.01, and ***p < 0.001 were considered significant.

## Results

### MiR-125b-5p is Decreased in Pan-Cancer

**FIGURE 1:**
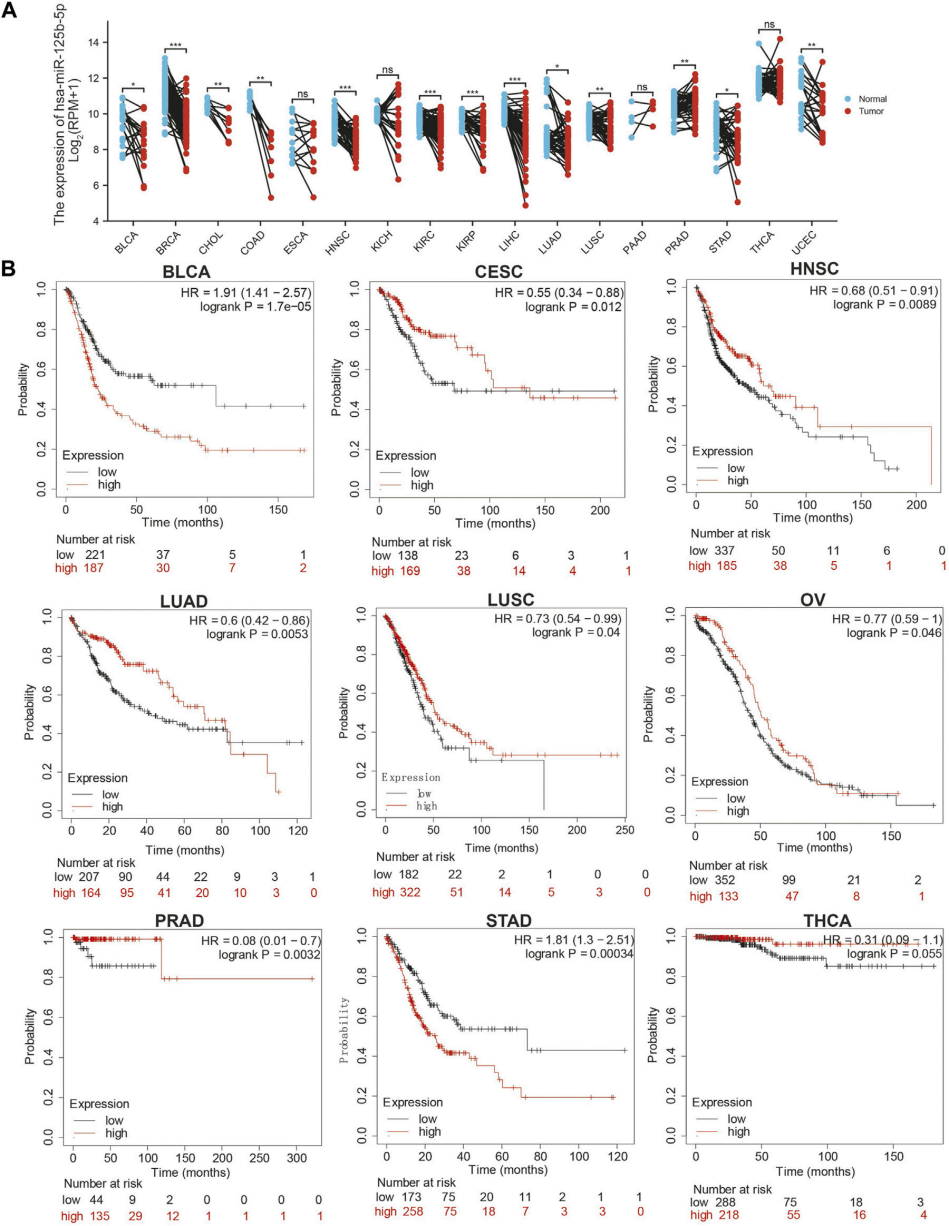
Expression analysis for miR-125b-5p in human cancers. **(A)** Expression of miR-125b-5p in pan-cancer. **(B)** Prognosis of miR-125b-5p in pan-cancer examined using the Kaplan–Meier plotter database.

**FIGURE 2:**
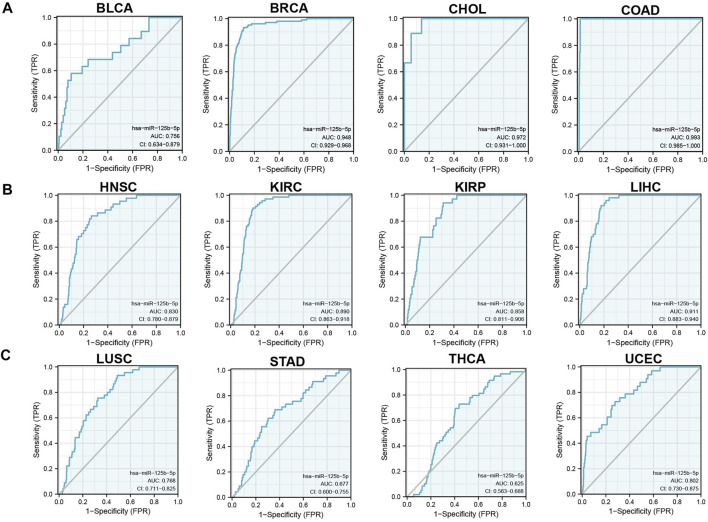
Analysis of the receiver operating characteristic (ROC) curve for miR-125b-5p in human cancers. **(A–C)** ROC curve analysis and area under the curve (AUC) values for miR-125b-5p in diverse human cancers.

We initially examined the expression of miR-125b-5p in multiple cancer types using the TCGA database. The results showed that the expression of miR-125b-5p was significantly lower in breast invasive carcinoma, bladder urothelial carcinoma, cholangiocarcinoma, colon adenocarcinoma, head and neck squamous cell carcinoma, kidney renal clear cell carcinoma, kidney renal papillary cell carcinoma, liver hepatocellular carcinoma, lung adenocarcinoma, lung squamous cell carcinoma, stomach adenocarcinoma, and uterine corpus endometrial carcinoma. On the contrary, miR-125b-5p showed a significantly higher expression in prostate adenocarcinoma (Figure 1A). These data confirmed that miR-125b-5p mainly decrease in multiple cancer types. Furthermore, we analyzed the prognostic value of miR-125b-5p in pan-cancer. The results demonstrated that a low expression of miR-125b-5p was correlated with poor prognosis in cervical squamous cell carcinoma and endocervical adenocarcinoma, head and neck squamous cell carcinoma, lung adenocarcinoma, lung squamous cell carcinoma, ovarian serous cystadenocarcinoma, prostate adenocarcinoma, and thyroid carcinoma. A low expression of miR-125b-5p was also correlated with better prognosis in bladder urothelial carcinoma and stomach adenocarcinoma (Figure 1B). Given that miR-125b-5p significantly affected the prognosis of multiple cancer types, therefore, we next examined its diagnostic value in human cancer patients. Receiver operating characteristic (ROC) curve analysis demonstrated higher sensitivity and specificity of miR-125b-5p for detection in pan-cancer (Figure 2). These data support the potential of miR-125b-5p as a biomarker in multiple cancer types.

### Analysis of the Function of the Target Genes of miR-125b-5p

**FIGURE 3:**
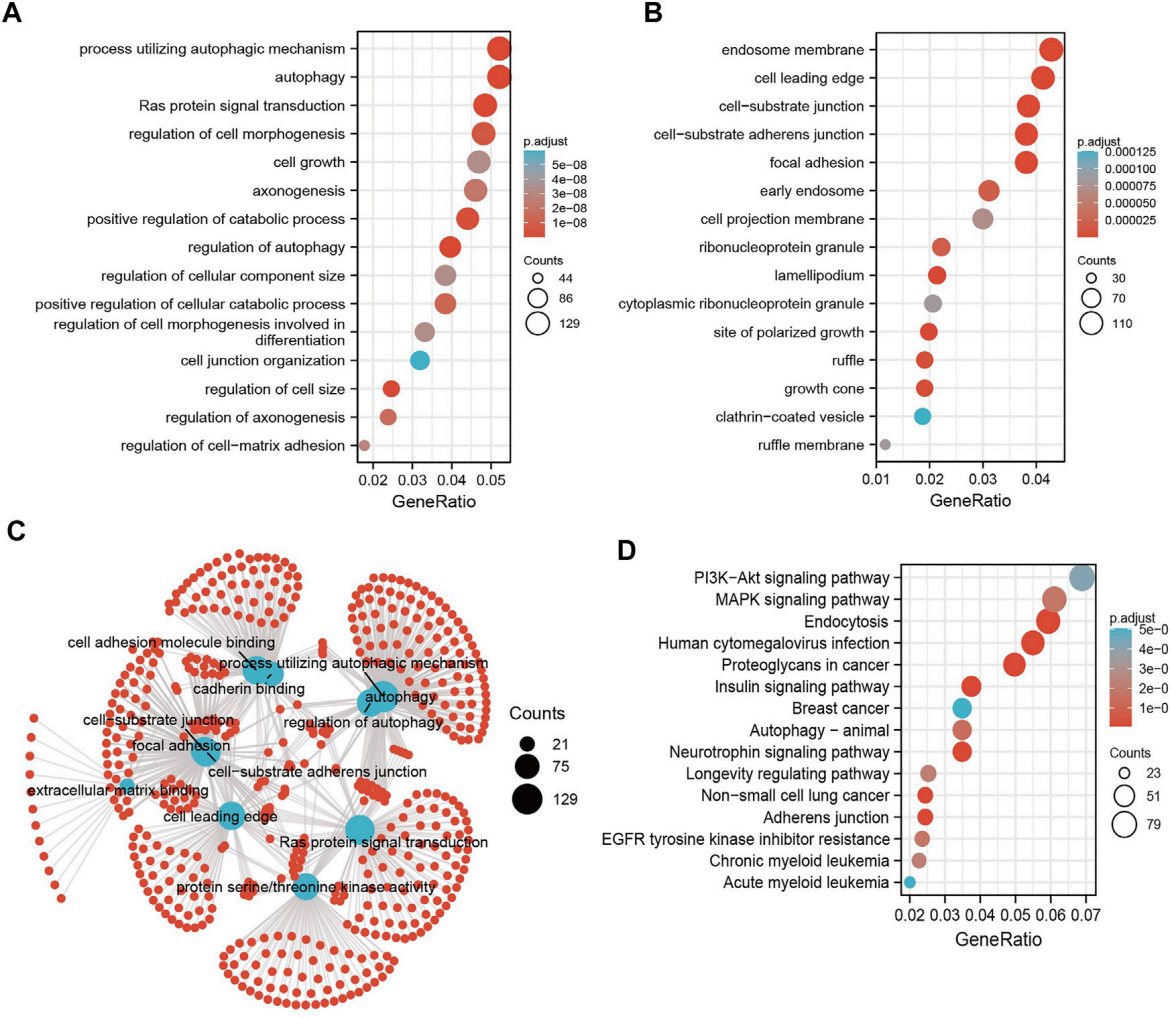
Analysis of the functions of the downstream target genes of miR-125b-5p in human cancers. **(A–C)** Gene Ontology (GO) terms of the downstream target genes of miR-125b-5p in pan-cancer analysis. **(D)** Kyoto Encyclopedia of Genes and Genomes (KEGG) signaling pathways of the downstream target genes of miRNA-125b-5p in pan-cancer analysis by starBase.

MicroRNAs mainly regulate the expression of messenger RNAs (mRNAs) and play an important role in diverse cancer progression. Therefore, we utilized starBase to predict the target genes of miR-125b-5p and used these genes to perform Gene Ontology (GO) and Kyoto Encyclopedia of Genes and Genomes (KEGG) analyses. The top 16 significant terms in the enrichment analysis of biological process (BP), molecular function (MF), and cellular component (CC) were presented. Notably, in terms of BP, the target genes of miR-125b-5p were enriched in the regulation of autophagy, Ras protein signal transduction, autophagy, process utilizing autophagic mechanism, regulation of cell size, positive regulation of catabolic process, regulation of cell morphogenesis, positive regulation of cellular catabolic process, regulation of axonogenesis, axonogenesis, regulation of cell–matrix adhesion, regulation of cell morphogenesis involved in differentiation, cell growth, regulation of cellular component size, cell junction organization, and endomembrane system organization (Figure 3A).

In terms of CC, the target genes of miR-125b-5p were enriched in the cell leading edge, focal adhesion, cell–substrate junction, cell–substrate adherens junction, endosome membrane, lamellipodium, site of polarized growth, growth cone, ruffle, early endosome, ribonucleoprotein granule, cell projection membrane, ruffle membrane, cytoplasmic ribonucleoprotein granule, and clathrin-coated vesicles (Figure 3B). In terms of MF, the target genes of miR-125b-5p were enriched in cell adhesion molecule binding, extracellular matrix binding, protein serine/threonine kinase activity, cadherin binding, mitogen-activated protein kinase binding, Ras GTPase binding, miRNA binding, regulatory RNA binding, modification-dependent protein binding, small GTPase binding, and p53 binding (Figure 3C). Furthermore, the top 15 KEGG pathways for the target genes of miR-125b-5p were enriched in human cytomegalovirus infection, endocytosis, neurotrophin signaling pathway, adherens junction, insulin signaling pathway, non-small cell lung cancer, proteoglycans in cancer, autophagy—animal, MAPK signaling pathway, EGFR tyrosine kinase inhibitor resistance, longevity regulating pathway, chronic myeloid leukemia, PI3K/Akt signaling pathway, and breast cancer and acute myeloid leukemia (Figure 3D).

### Analysis of the Correlation Between miR-125b-5p Expression and Clinicopathological Characteristics in LUAD

**FIGURE 4:**
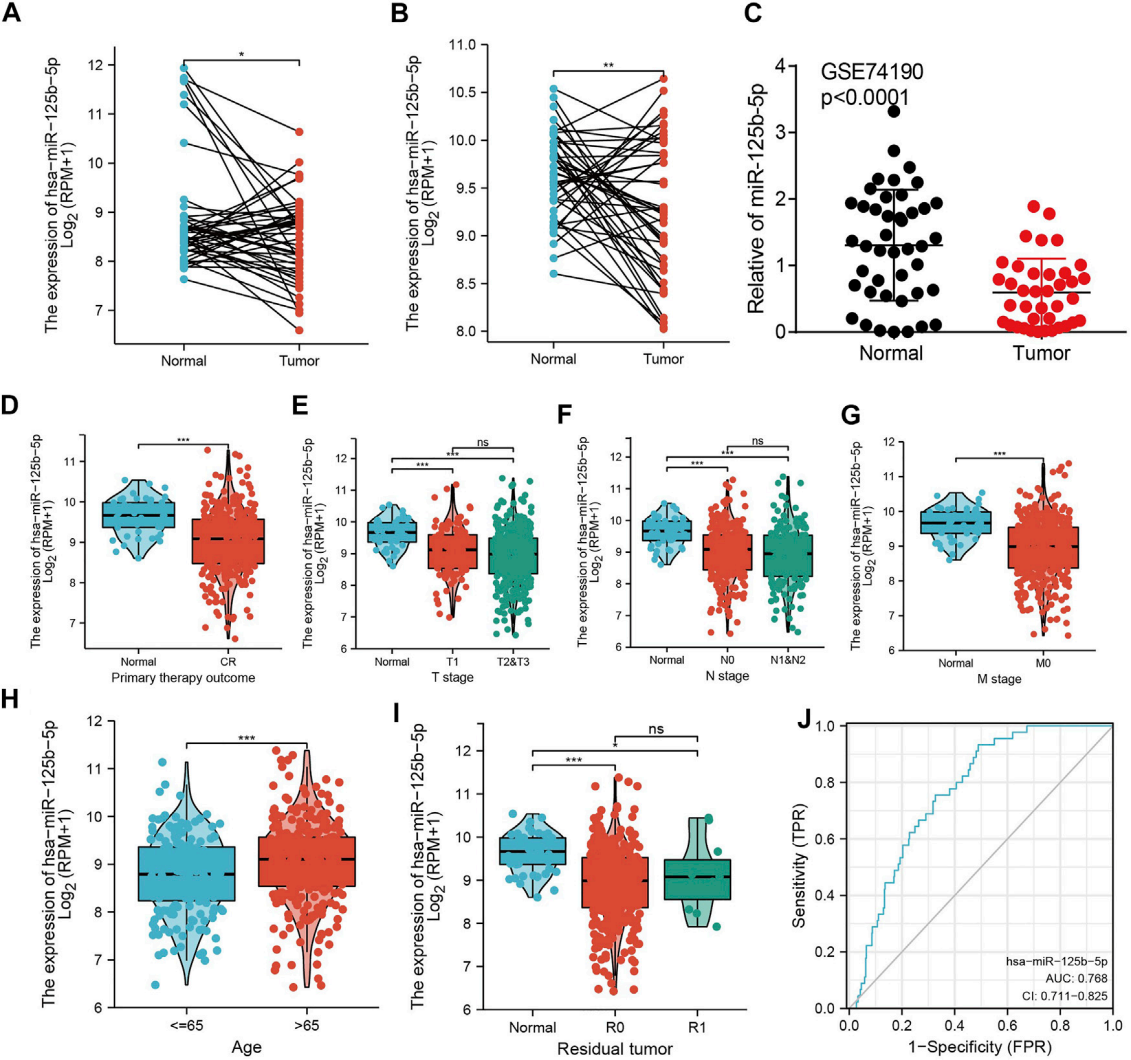
miR-125b-5p was decreased in lung adenocarcinoma (LUAD). **(A–C)** Examination of the expression of miR-125b-5p in LUAD in The Cancer Genome Atlas (TCGA) and Gene Expression Omnibus (GEO) datasets. **(D–I)** Correlation between miR-125b-5p expression and clinicopathological characteristics of LUAD, including primary therapy outcome, TNM stage, age, and residual tumor. **(J)** Receiver operating characteristic (ROC) curve analysis and area under the curve (AUC) values for miR-125b-5p in LUAD. *p < 0.05, **p < 0.01, ***p < 0.001.

**FIGURE 5:**
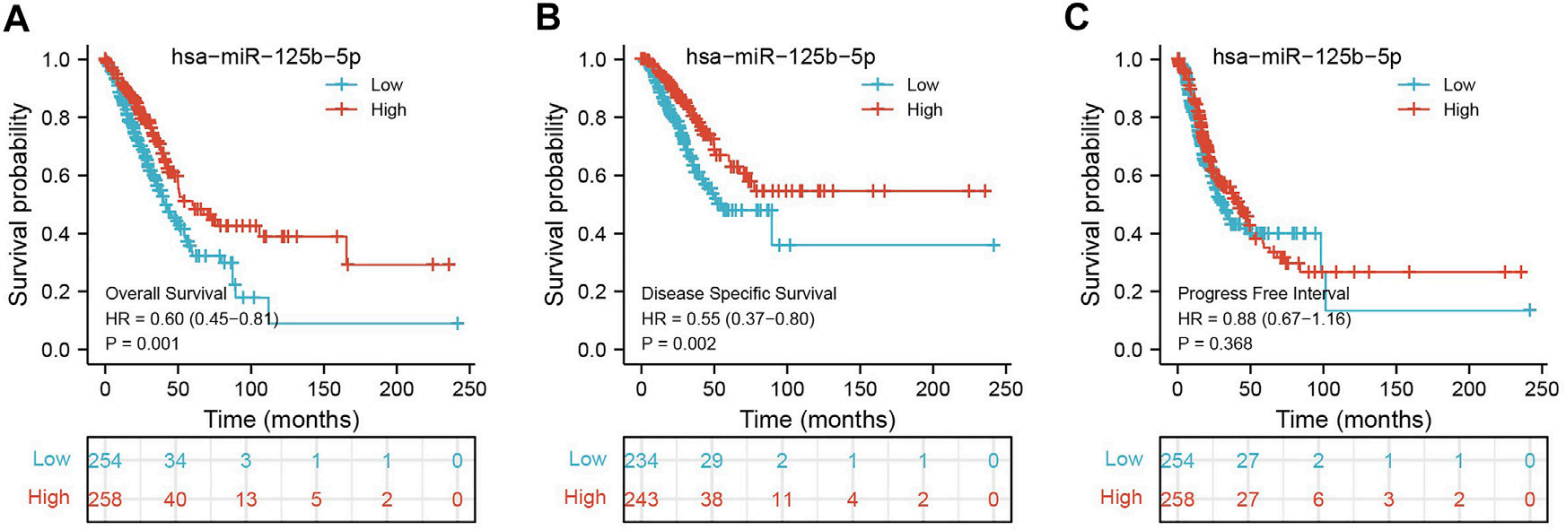
Prognostic value of miR-125b-5p in lung adenocarcinoma (LUAD). Prognostic value of miR-125b-5p in LUAD, including overall survival **(A)**, disease-specific survival **(B)**, and progression-free interval **(C)**.

To examine the expression of miR-125b-5p in LUAD, we analyzed the datasets from TCGA and Genetic Testing Ontology (GTO) and found that miR-125b-5p was decreased in LUAD tissues compared to that in normal tissues (Figures 4A–C). Furthermore, a lower expression correlated with poor clinicopathological features, including the primary therapy outcome, TNM stage, age, and residual tumor (Figures 4D–I). The ROC curve analysis of miR-125b-5p showed an area under the curve (AUC) value of 0.768 for LUAD patients (Figure 4J). Given that the expression of miR-125b-5p correlated with poor clinicopathological features in LUAD, therefore, we next explored the prognostic value of miR-125b-5p in non-small cell lung cancer. The results demonstrated that a lower expression of miR-125b-5p was significantly associated with poor overall survival and disease-specific survival (Figures 5A–C).

### Overexpression of miR-125b-5p Inhibits LUAD Progression

**FIGURE 6:**
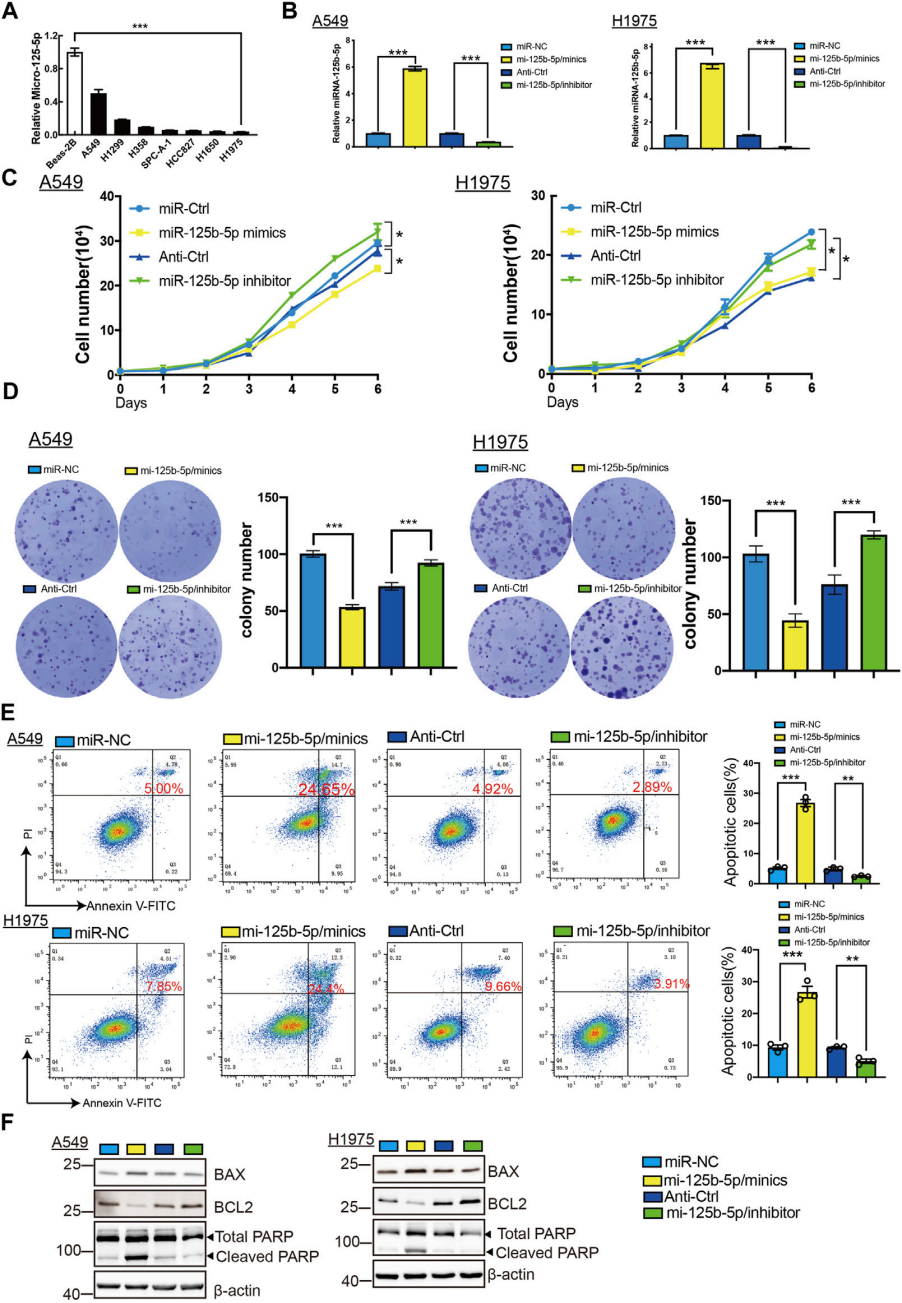
Overexpression of miR-125b-5p inhibited lung adenocarcinoma (LUAD) cell proliferation. **(A)** Expression of miR-125b-5p in LUAD cell lines examined by the quantitative real-time PCR (qRT-PCR) assay. **(B)** Expression of miR-125b-5p in LUAD cell lines after its overexpression examined using the qRT-PCR assay. **(C,D)** Overexpression of miR-125b-5p on cell growth ability examined using the growth curve and colony formation assays. Scale bar, 50 μm. **(E,F)** Overexpression of miR-125b-5p on cell apoptosis examined by flow cytometry and Western blot assay. Quantification data are also indicated. *p < 0.05, **p < 0.01, ***p < 0.001.

**FIGURE 7:**
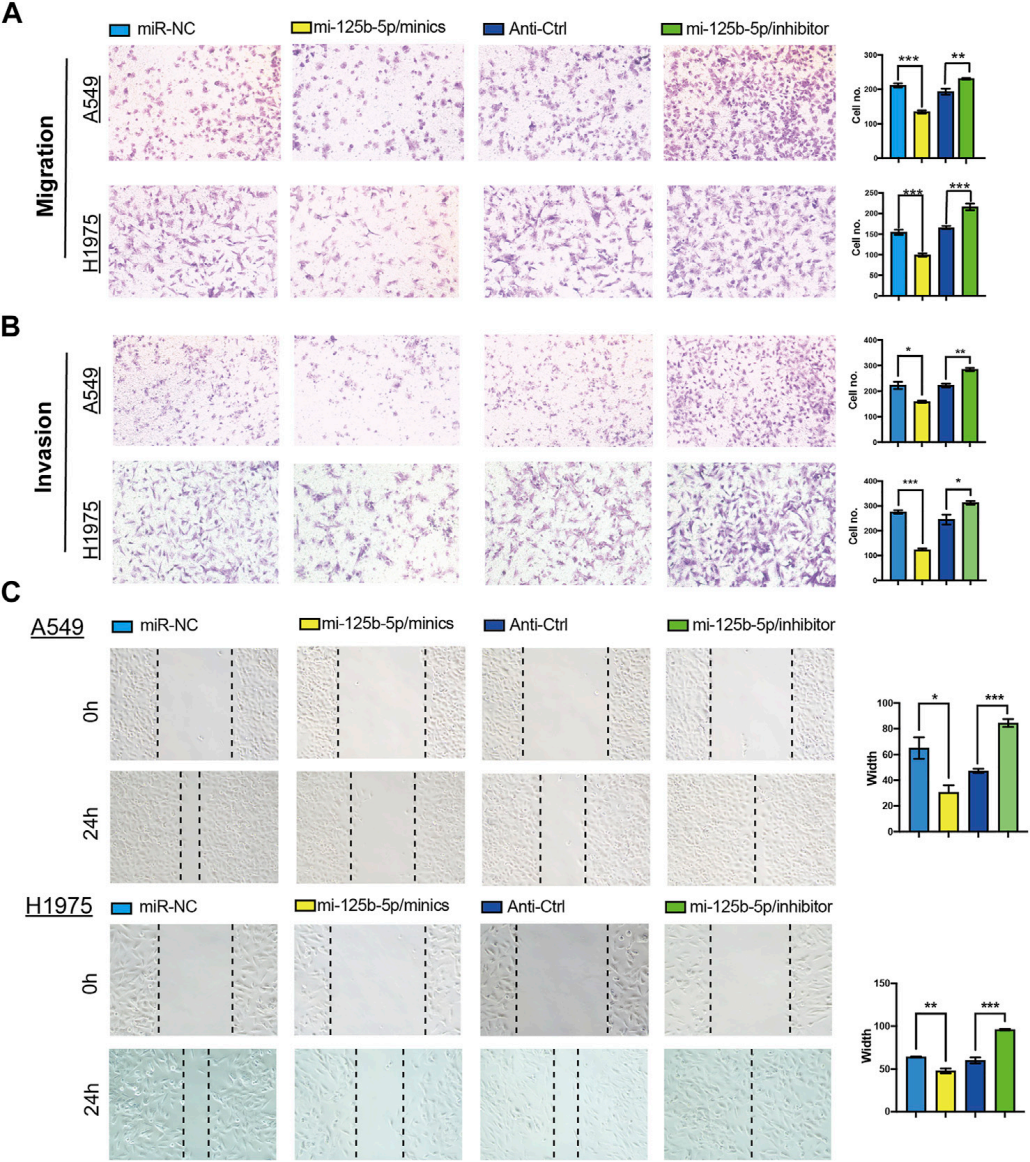
Overexpression of miR-125b-5p inhibited lung adenocarcinoma (LUAD) migration and invasion. **(A–C)** Overexpression of miR-125b-5p on cell migration and invasion abilities examined by the Transwell and wound healing assays. Quantification data are also indicated. *p < 0.05, **p < 0.01, ***p < 0.001.

To elucidate the biological functions of miR-125b-5p in LUAD cells, we transfected miR-125b-5p mimics and inhibitors in A549 and H1975 cells. The efficiency of miR-125b-5p knockdown and overexpression in A549 and H1975 cells was verified by the qRT-PCR assay (Figure 6B). Growth curve and colony formation assays were carried out to evaluate cell proliferation, with the results showing that the overexpression of miR-125b-5p notably inhibited the proliferation of A549 and H1975 cells, while miR-125b-5p knockdown inhibited increased cell growth (Figures 6C,D). Additionally, we found that the overexpression of miR-125b-5p promoted cell apoptosis, with miR-125b-5p inhibiting the reduction of cell apoptosis, which was further confirmed by Western blot examining the expression of marker genes critical for cellular apoptosis, such as Bax, Bcl-2, and PARP (Figure 6F). Finally, the Transwell and wound healing assay revealed that the overexpression of miR-125b-5p reduced the migration and invasion of LUAD cells, while miR-125b-5p knockdown had the opposite effect (Figures 7A,B). Taken together, these data suggest that miR-125b-5p exerts the role of a suppressor gene in LUAD cells.

### Correlation of miR-125b-5p and Immune Cells in LUAD

**FIGURE 8:**
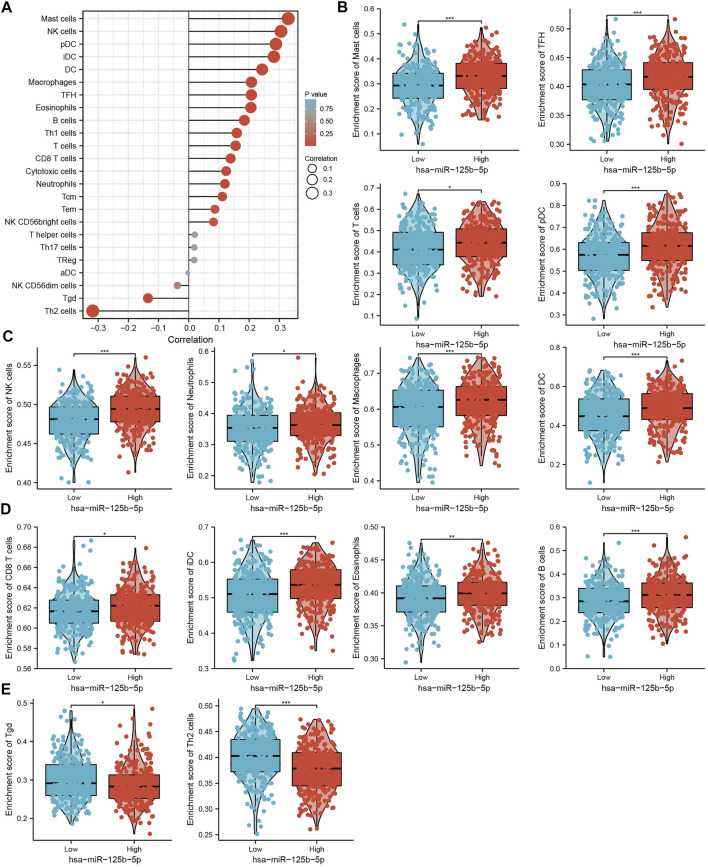
Analysis of the correlation between the expression of miR-125b-5p and immune infiltration. **(A)** Correlation between the relative abundance of 24 immune cells and the expression level of miR-125b-5p. **(B–E)** Diverse proportions of immune cell subtypes in tumor samples in high and low miR-125b-5p expression groups. *p < 0.05, **p < 0.01, ***p < 0.001.

We used ssGSEA to examine the correlation between miR-125b-5p and immune cell infiltration in LUAD. The results showed that miR-125b-5p was positively correlated with the infiltration levels of mast cells, natural killer (NK) cells, dendritic cells (DCs), plasmacytoid DCs (pDCs), interstitial DCs (iDCs), macrophages, T follicular helper (TFH) cells, eosinophils, B cells, type 1 T helper (Th1) cells, T cells, CD8 T cells, cytotoxic cells, neutrophils, and central memory (TCM) and effector memory (TEM) T cells (Figures 8A–E). However, Ted and Th2 cells were all negatively correlated with miR-125b-5p expression (Figures 8A–E). These results demonstrate that miR-125b-5p plays a significant role in the immune response of LUAD.

## Discussion

Lung cancer has become the leading cause of cancer-related deaths worldwide, with a rising trend of incidence and mortality. LUAD accounts for 85% of all lung cancers (Reck et al., 2014). It has been shown that miRNAs play a major role in regulating the occurrence and progression of cancer (Lee and Cheah, 2019). For instance, Qu et al. found that miR-133b had a low expression in renal cell carcinoma. The overexpression of miR-133b significantly inhibits cell proliferation, migration, and invasion of renal cell carcinoma. Research on its mechanism showed that miR-133b exerts this role *via* modulating the expression of MMP9 and inhibiting the progression of renal cell carcinoma (Wu et al., 2014). Furthermore, Zhang et al. showed that miR-125b targeted MMP11 and breast cancer progression (Wang et al., 2020). A previous study also showed that miR-125b inhibited bladder cancer cell proliferation and migration by targeting SphK1 (Zhao et al., 2015). However, the clinical significance and functional role of miR-125b-5p in LUAD remain unclear.

In the current study, we demonstrated that the expression levels of miR-125b-5p were significantly downregulated in LUAD cells compared with normal bronchial epithelial cells. The low expression of miR-125b-5p correlated with poor overall survival, disease-specific survival, and poor clinicopathological features, including the primary therapy outcome, TNM stage, age, and residual tumor. The ROC curve analysis of miR-125b-5p showed an AUC value of 0.768 for LUAD patients. These results indicate that miR-125b-5p has great potential as a detection index for the diagnosis of LUAD with high sensitivity and specificity.

For the function of miR-125b-5p in LUAD, we performed KEGG enrichment analysis and found that the target genes of miR-125b-5p were enriched in human cytomegalovirus infection, endocytosis, neurotrophin signaling pathway, adherens junction, insulin signaling pathway, non-small cell lung cancer, proteoglycans in cancer, autophagy animal, MAPK signaling pathway, EGFR tyrosine kinase inhibitor resistance, longevity regulating pathway, chronic myeloid leukemia, PI3K/Akt signaling pathway, and breast cancer and acute myeloid leukemia. These results suggest that miR-125b-5p may play an important role in the progression of LUAD.

Previous studies reported that miR-125b played a suppressor role in diverse cancer progression. For example, miR-125b regulated HMGA1 and inhibited cervical cancer progression. In this study, we found that miR-125b-5p knockdown promoted the proliferation, migration, and invasion of LUAD cells. Additionally, we found that knockdown of miR-125b-5p inhibited cell apoptosis, but its overexpression showed the opposite effect and promoted cell apoptosis, which was further confirmed by Western blot examining the expression of marker genes critical for cellular apoptosis, such as Bax, Bcl-2, and PARP. These results indicate that miR-125b-5p may inhibit the proliferation, migration, and invasion of LUAD cells. Above all, these results show that miR-125b-5p plays a suppressor role in LUAD cancer progression.

We further studied the immune role of miR-125b-5p in the tumor microenvironment. We analyzed the correlation between the expression of miR-125b-5p and diverse immune cell infiltration in LUAD. The results showed that miR-125b-5p was positively correlated with the infiltration levels of mast cells, NK cells, pDCs, iDCs, DCs, macrophages, TFH cells, eosinophils, B cells, Th1 cells, T cells, CD8 T cells, cytotoxic cells, neutrophils, TCM, and TEM. These results at least partially confirm that miR-125b-5p plays a crucial role in immune response regulation.

## Conclusion

In summary, we demonstrated that miR-125b-5p plays a significant role in the progression of LUAD. This study has thus provided a new perspective in the research on miRNAs in LUAD, as well as a scientific basis for novel developments in the diagnosis and treatment of LUAD.

## Data Availability

The original contributions presented in the study are included in the article/Supplementary Material, Further inquiries can be directed to the corresponding authors.
